# Supplementary material for: Variability in Codon Usage in Coronaviruses Is Mainly Driven by Mutational Bias and Selective Constraints on CpG Dinucleotide
Source: Viruses. 2021 Sep 10;13(9):1800. doi: 10.3390/v13091800 (PMC8473333; doi:10.3390/v13091800)
Supplement: Supplementary file 1 [file viruses-13-01800-s001.zip › figureSubmission/figures_sup.pdf]

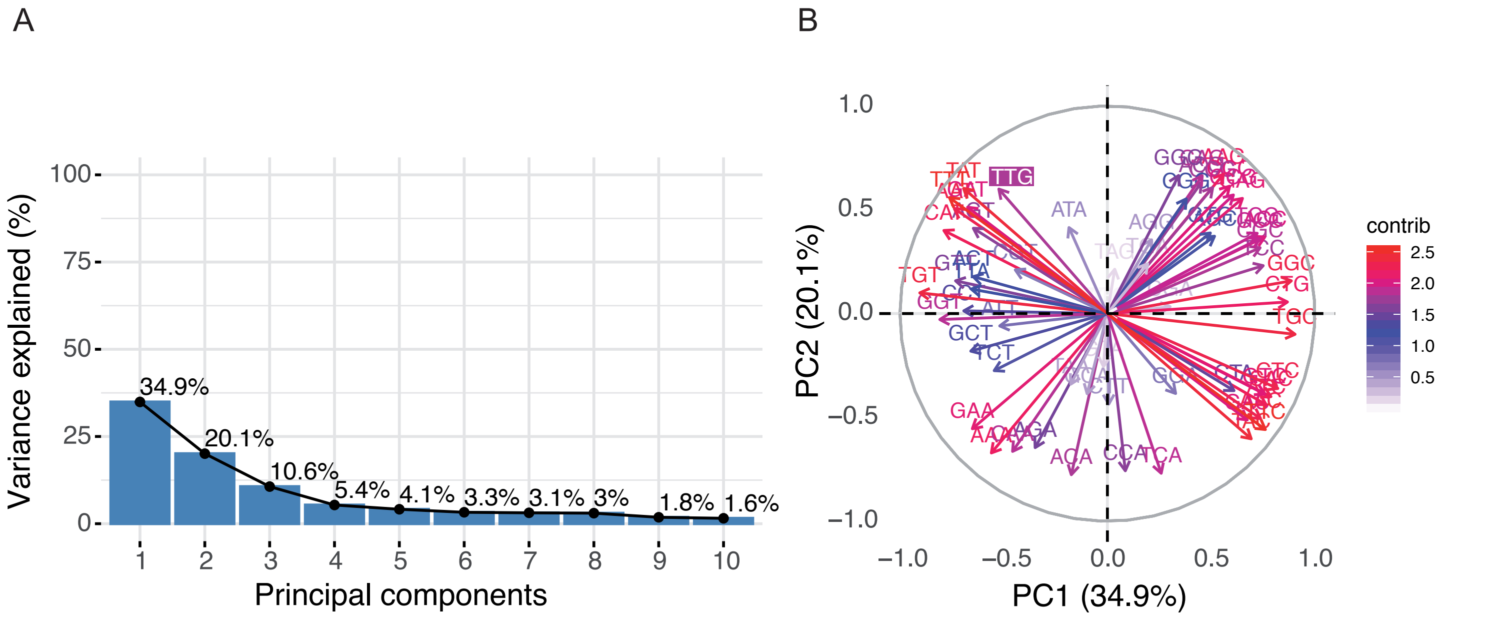


**Supplementary Figure 1**: (A) Bar chart representing the percentage of variance explained by each principal component. (B) Projection of the individual codon on the first two principal components.

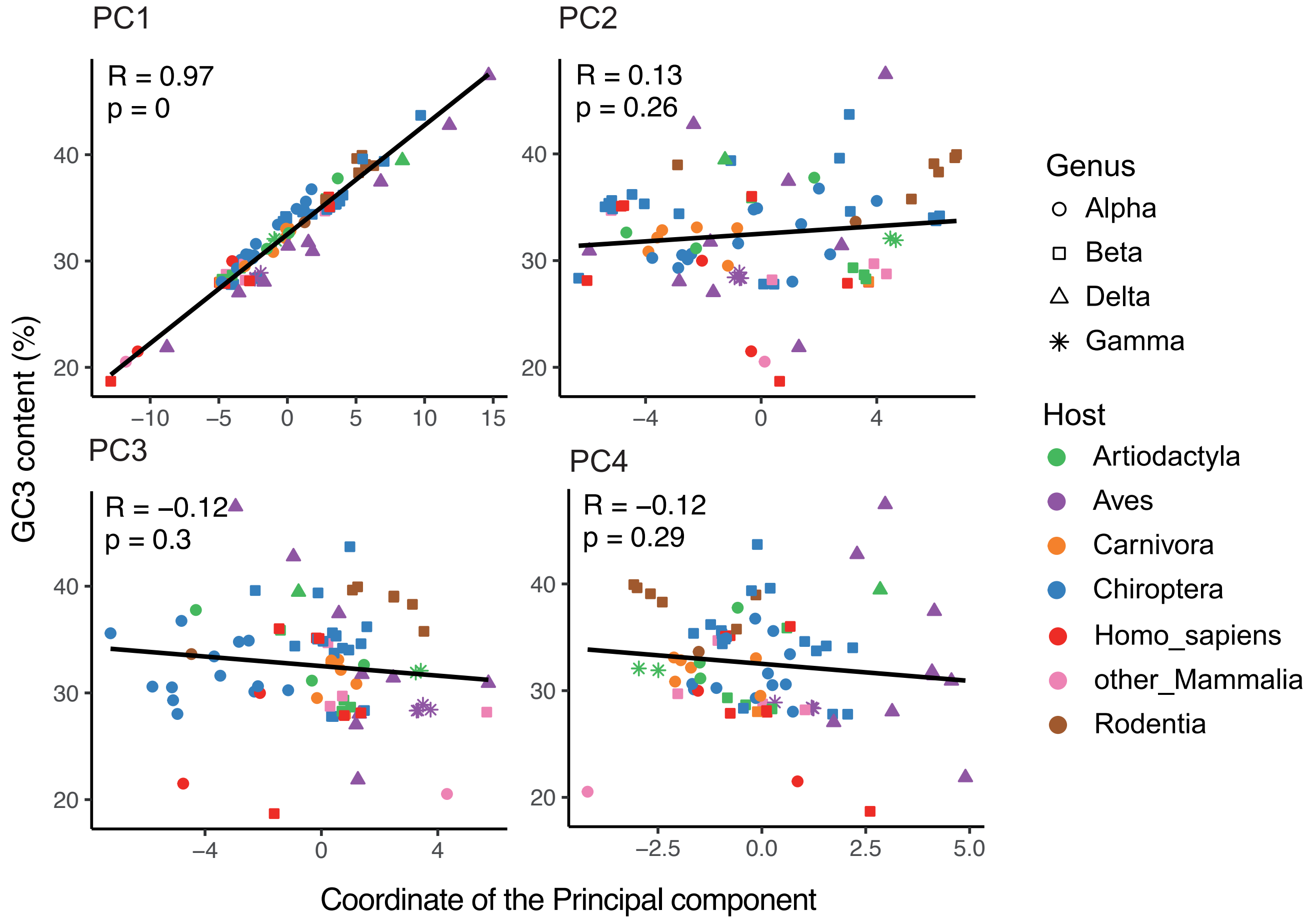


**Supplementary Figure 2**: Spearman correlation between GC3 content (%) and coordinate of the first 4 principal components.

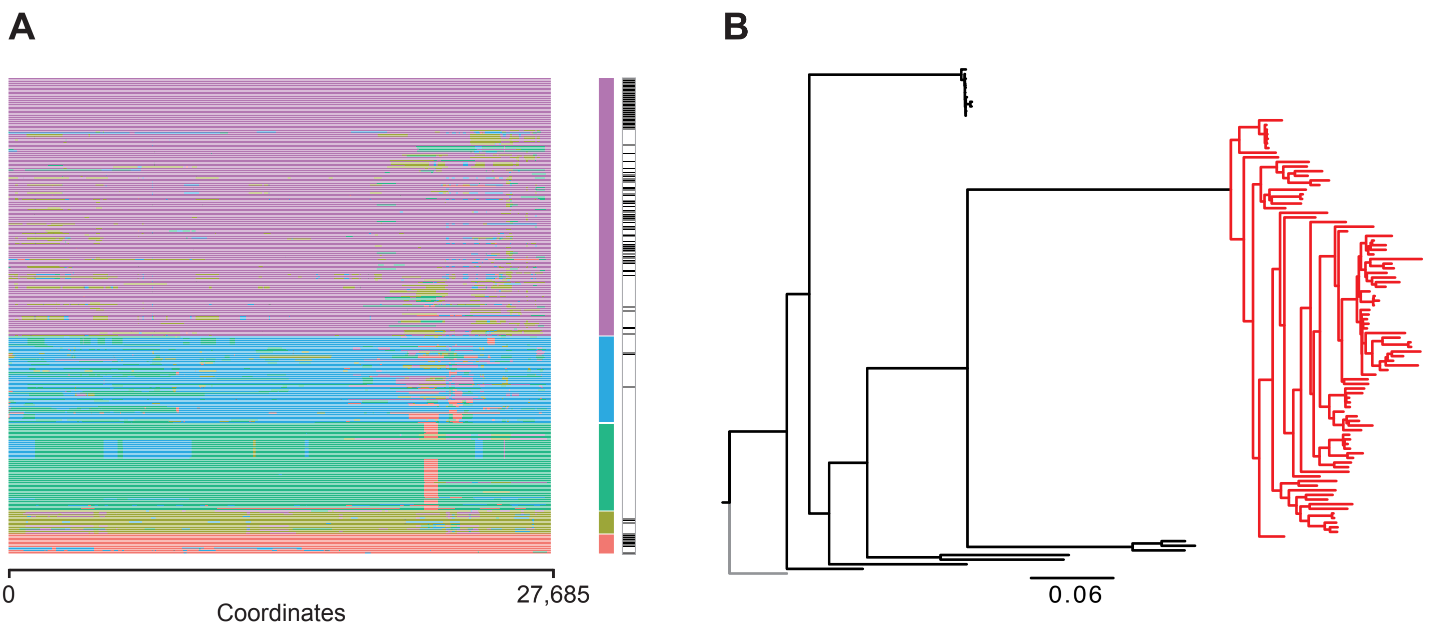


**Supplementary Figure 3:** Representation of the steps we used to select strains for our metapopulation analysis through the example of the infectious bronchitis virus. (A) Representation of the recent recombinations events happening within strains inferred by fastGear. Rows correspond to viral strains sequences, the columns to positions in the alignment, and colors show the membership of each portion of sequences to lineage detected by fastGear. Sequence colored in black on the left side represent sequence selected to generate the phylogenetic tree. (B) Phylogenetic tree of the relationship among viral strains. The tree is rooted based on an outgroup (grey) as indicated on Supplementary Table 2.

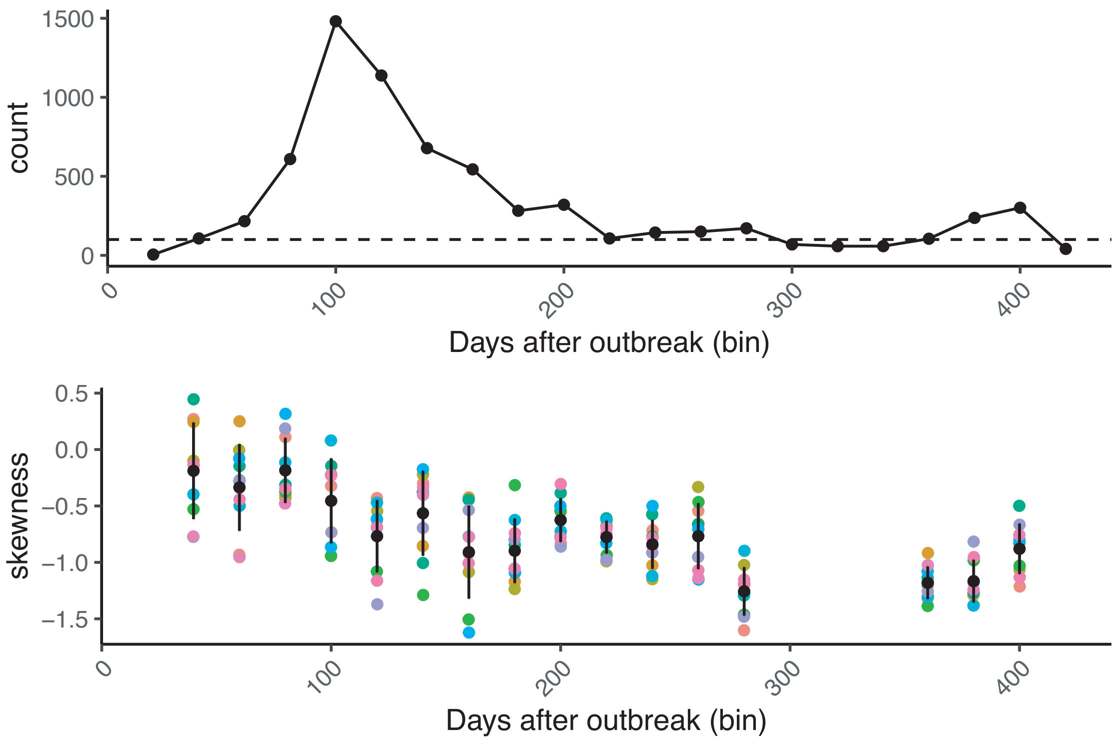


**Supplementary Figure 4**: Number of SARS-CoV2 sequenced genomes since the beginning of the outbreak, represented by bin of 10,000 sequences (top panel). Estimation of the SFS skweness, by subsampling 5 replicate of 1,000 sequences within each bin (bottom panel).

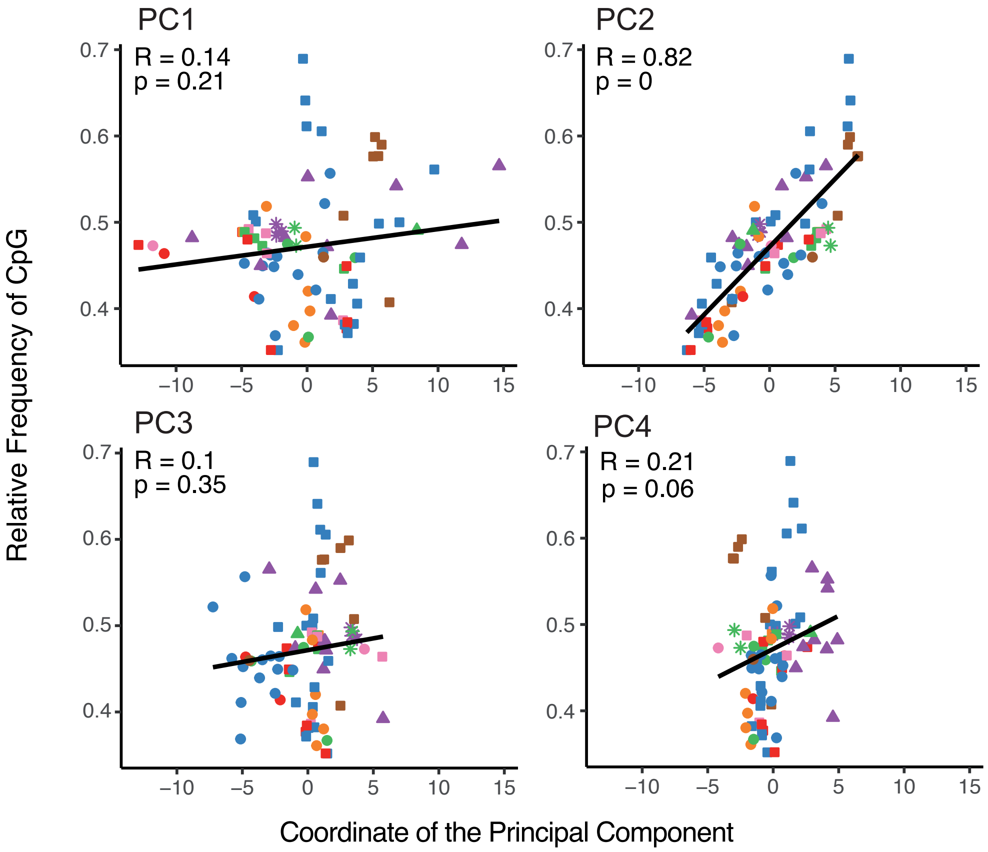


**Supplementary Figure 5:** Spearman correlation between relative abundance of CpG dinucleotide and coordinate of the first 5 principal components

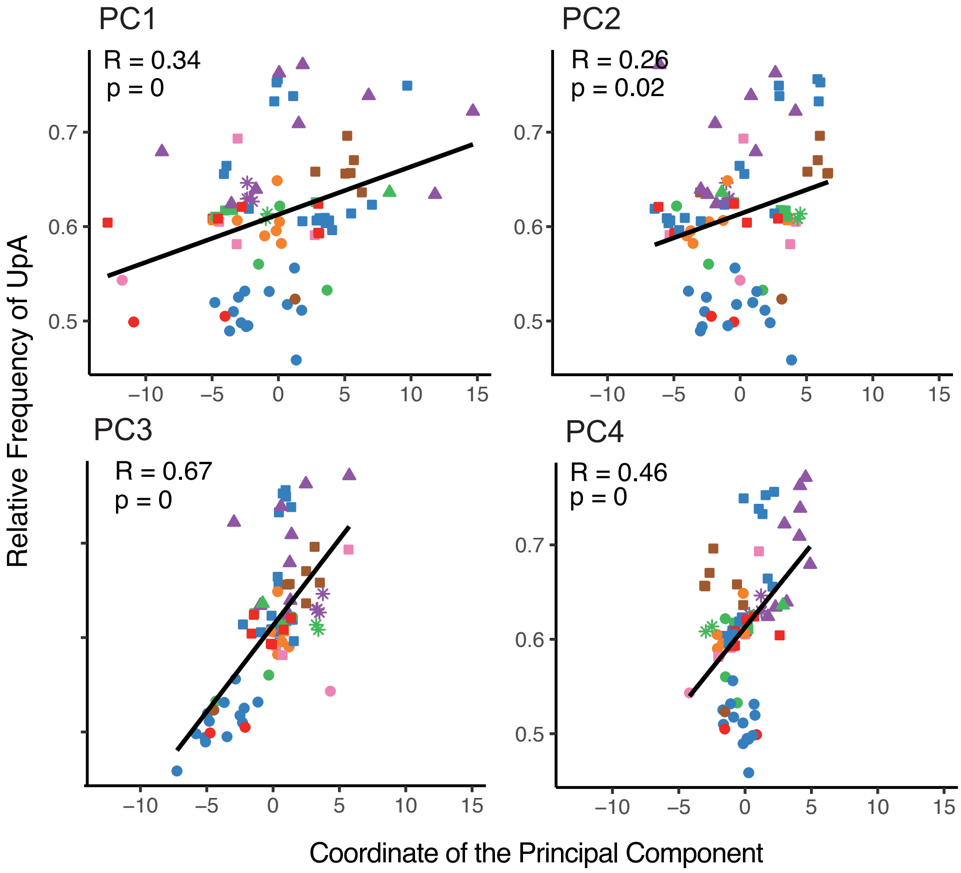


**Supplementary Figure 6:** Spearman correlation between relative abundance of UpA dinucleotide and coordinate of the first 4 principal components.

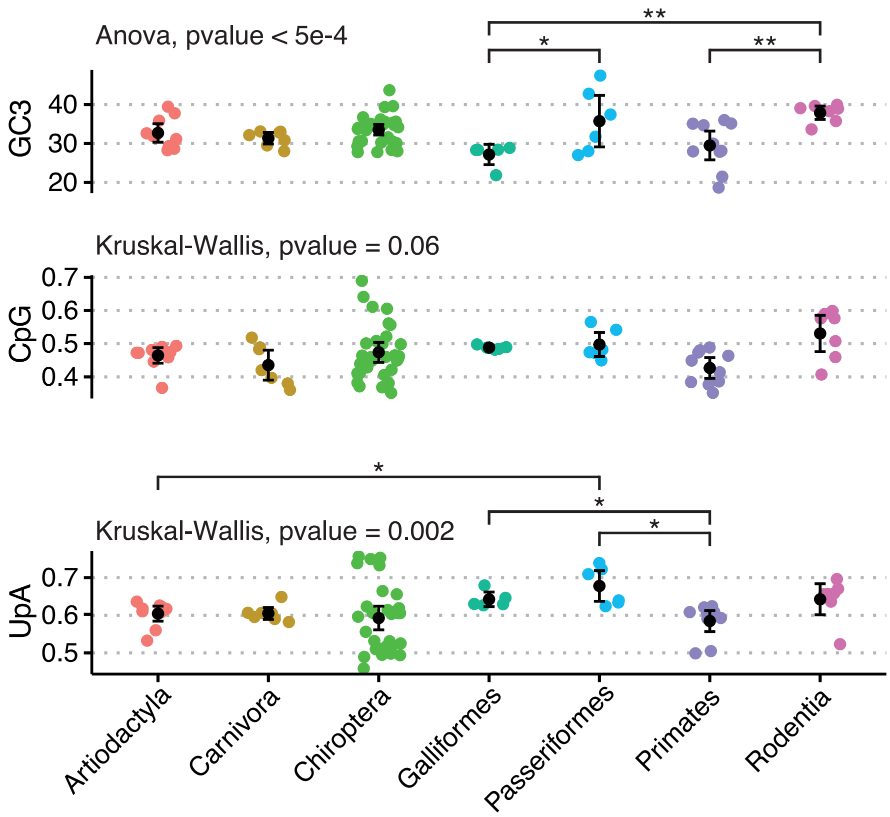


**Supplementary Figure 7:** Comparison of the CG3 content, CpG, and UpA dinucleotide relative abundances across CoVs hosts. Variance analysis of normally distributed data was done using ANOVA follow by a post doc Tukey test showing further individual pairwise differences. Non-normal data were processed using Kruskal-Wallis test follow by a rank Wilcoxon test with a Bonferroni correction (code for p-value: * <0.05, ** <0.01, *** <0.001).

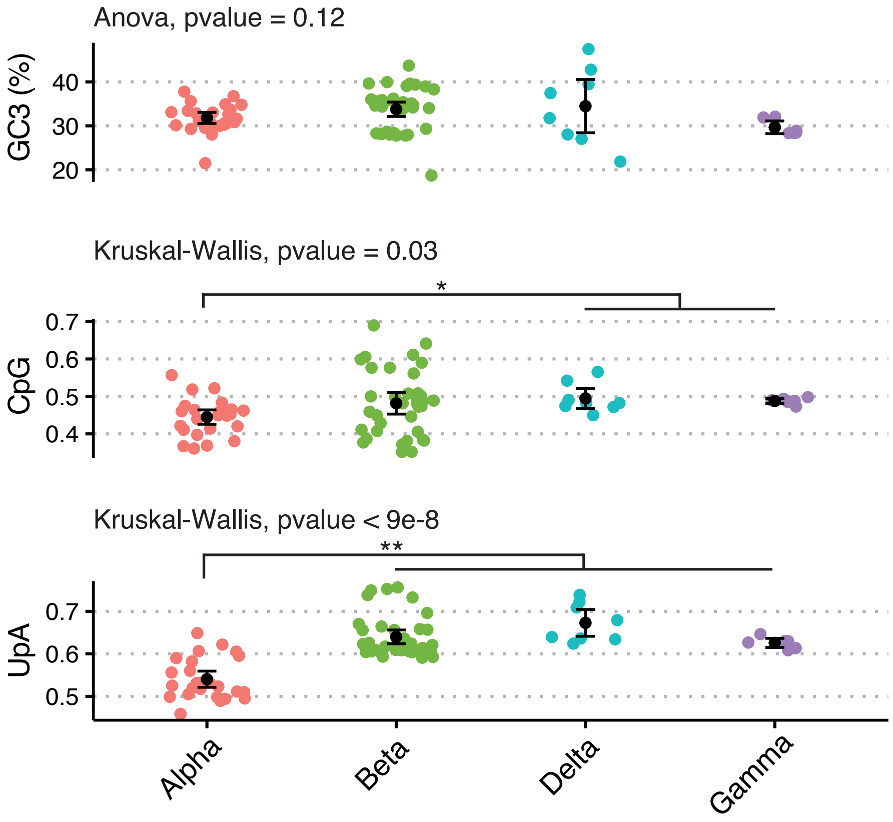


**Supplementary Figure 8**: Comparison of the CG3 content, CpG, and UpA dinucleotide relative abundances across CoVs genera. Statistical significance has been treated similarly than above.
