# Supplementary figures and images for: Variability in Codon Usage in Coronaviruses Is Mainly Driven by Mutational Bias and Selective Constraints on CpG Dinucleotide
Source: Viruses. 2021 Sep 10;13(9):1800. doi: 10.3390/v13091800 (PMC8473333; doi:10.3390/v13091800)

**A**

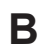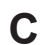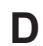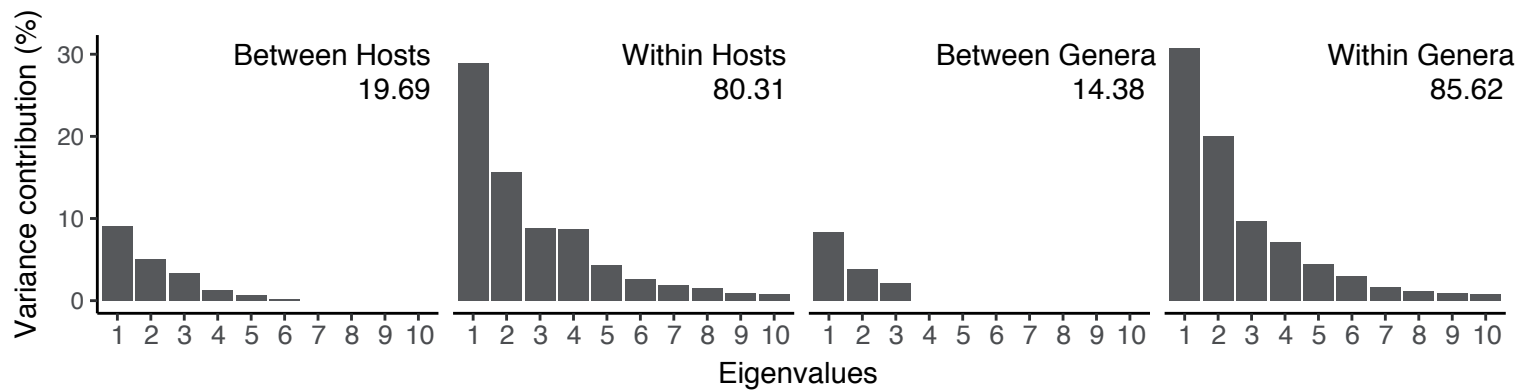

Figure 2

A

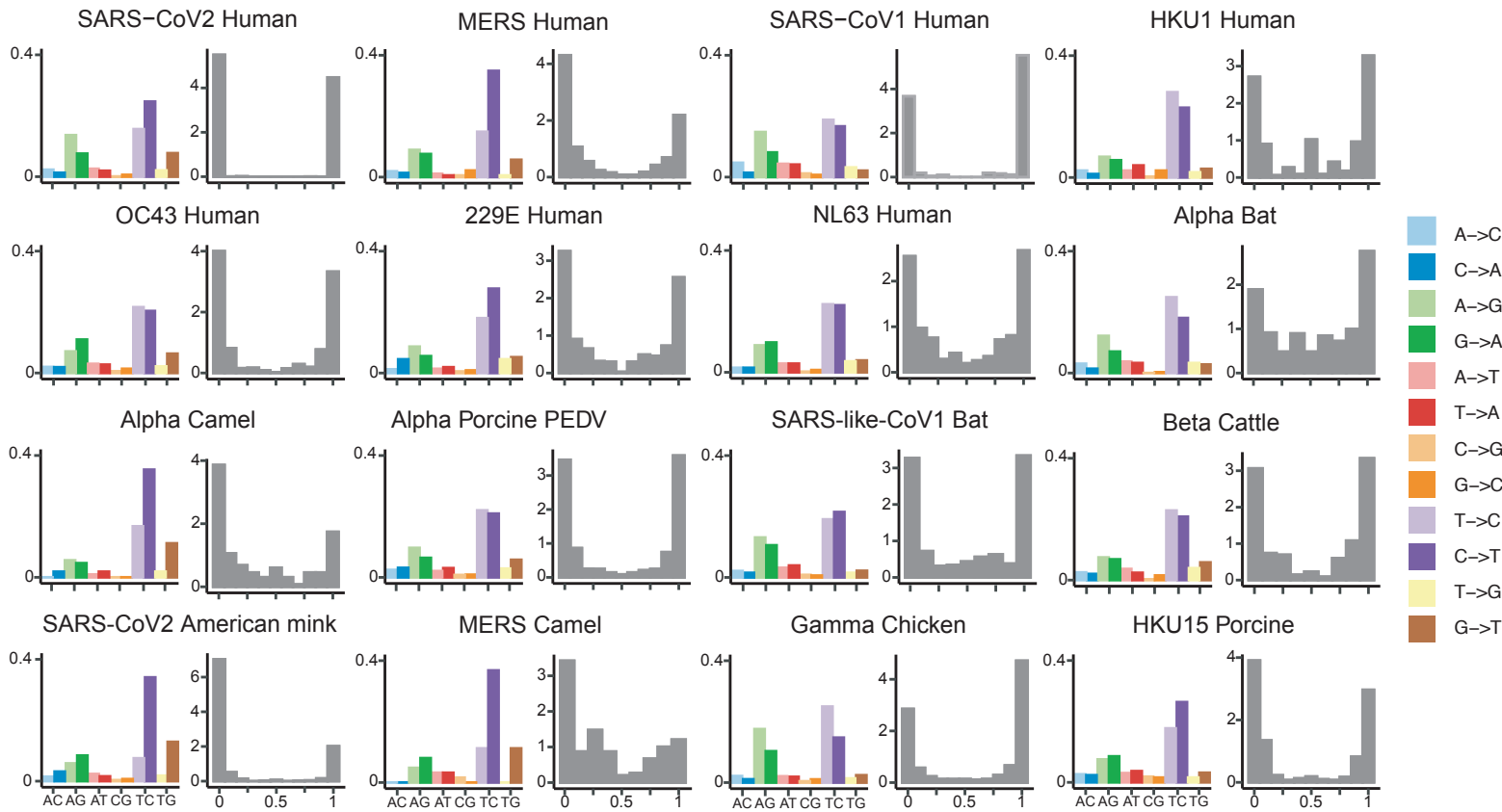

B

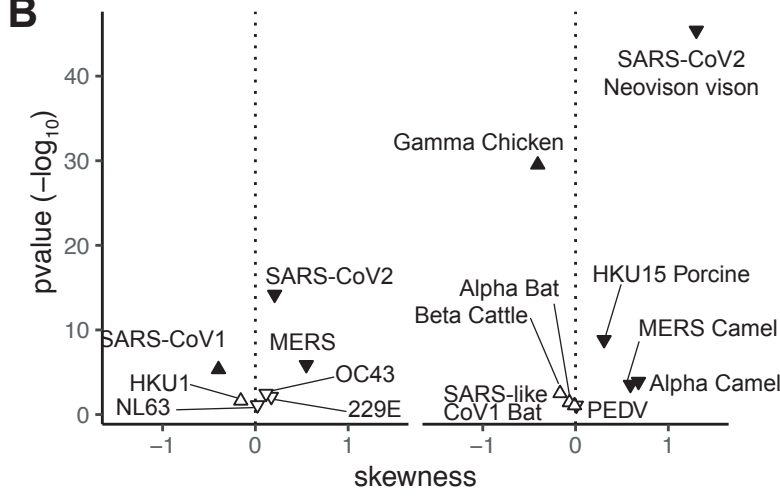

C

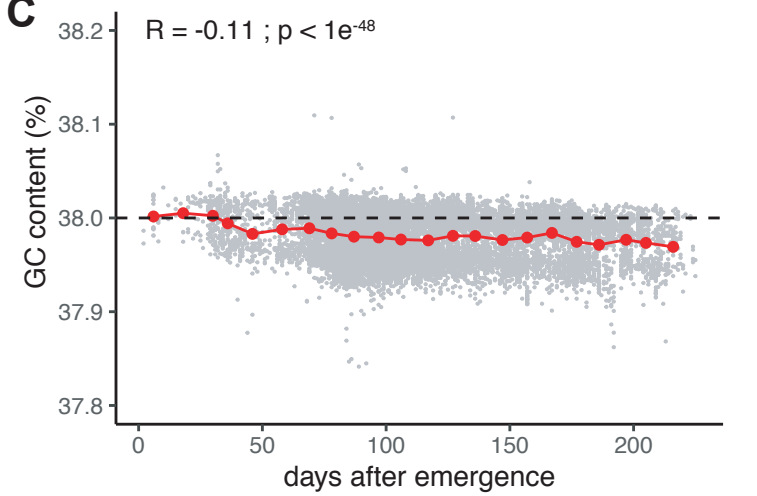

**Figure 3****A**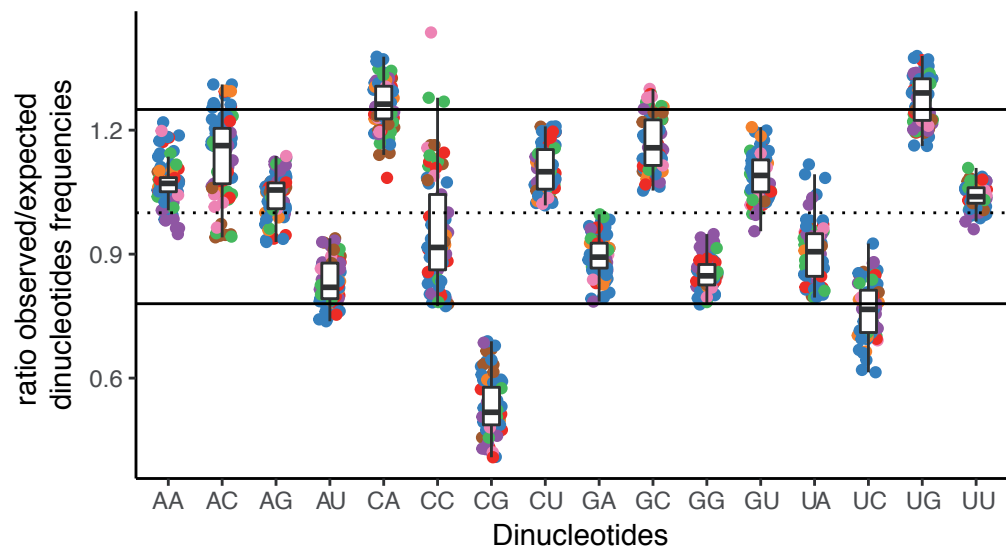**B**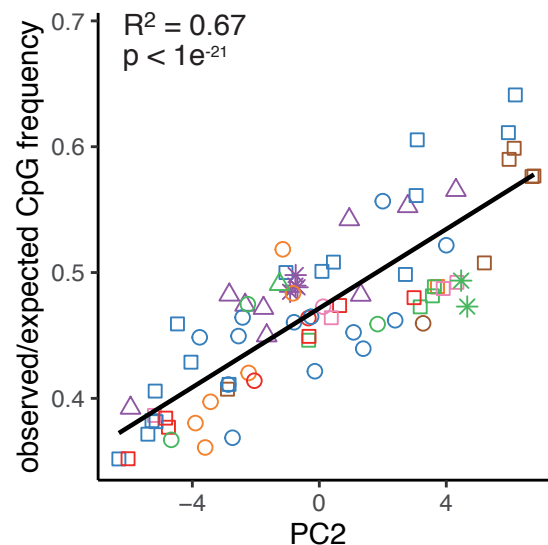**C**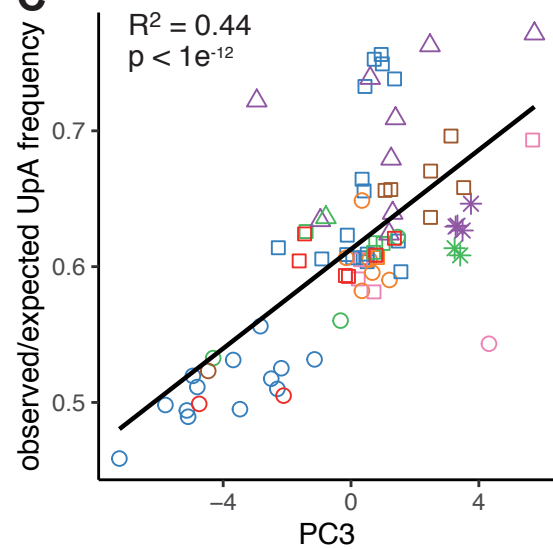**D**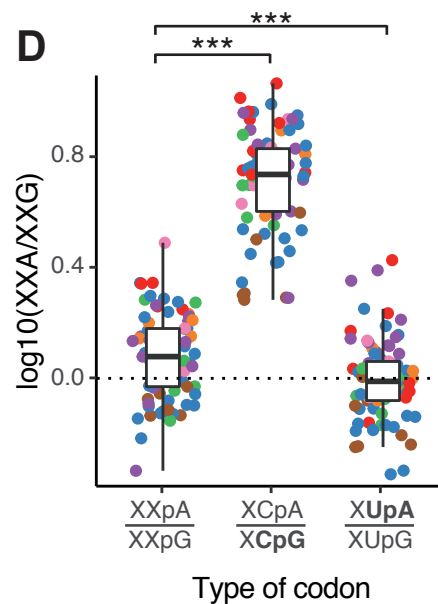**E**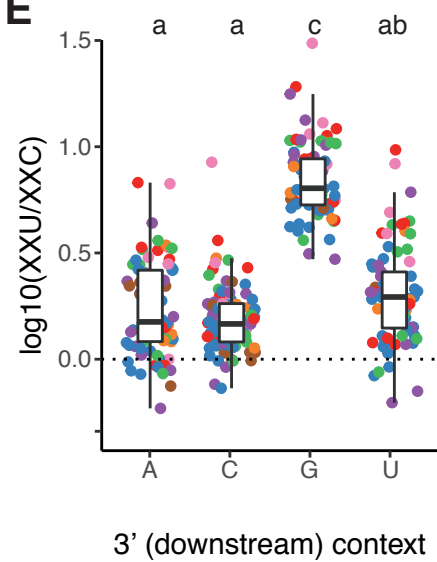

Figure 4

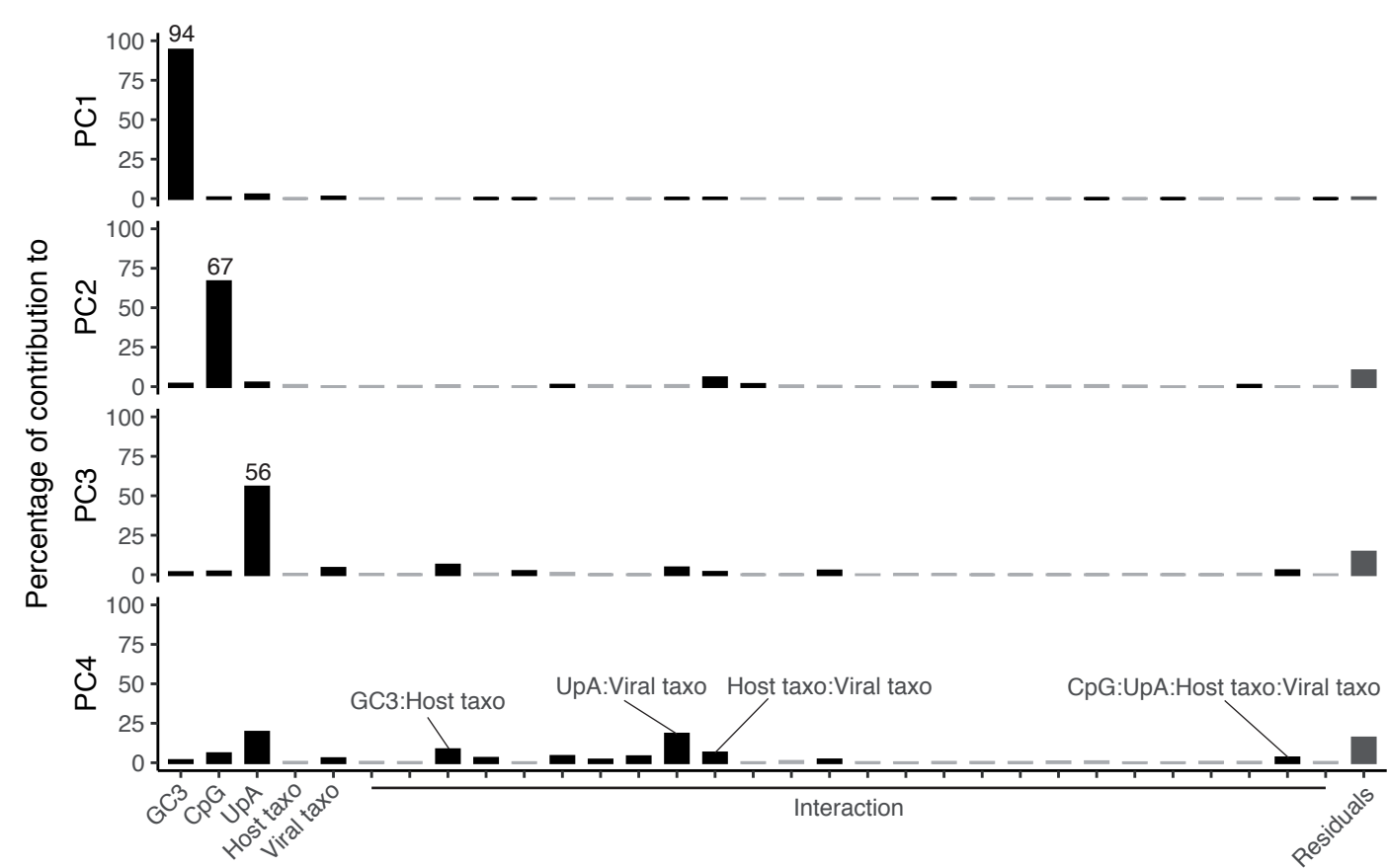

Supplement: Supplementary file 1 [file viruses-13-01800-s001.zip › figureSubmission/figures.pdf]
